# The Freeway Running through the Yard: Traffic Exhaust and Asthma in Children

**Published:** 2006-05

**Authors:** Carol Potera

Since its inception 13 years ago, the Children’s Health Study has indicated that air pollution in Southern California communities reduces lung growth and development, raises the risk of developing asthma, and increases school absences due to respiratory illnesses. The latest finding from the study team zeroes in on the impact of exposure to traffic-related pollutants at home, and shows that kindergarten and first-grade students who lived near busy roads experienced a higher prevalence of asthma **[*EHP* 114:766–772; McConnell et al.]**.

The researchers evaluated the respiratory health of 5,341 children relative to the distance that they lived from major roads, including highways, arterial roads, and freeways. The children, aged 5 to 7 years, lived in 13 communities. The team used detailed information about roadway type and traffic volume collected by the California Department of Transportation to develop a proxy for fresh traffic exhaust—the gases given off immediately around cars—at each child’s home.

Children who lived within 75 meters of a major road (about the length of a city block) were approximately 1.5 times more likely to report asthma or wheezing compared to those living 300 meters or more from a major road. Among children with no parental history of asthma, those who had resided at an address close to heavy traffic since before age 2 experienced even higher risks (2.5-fold for asthma and 2.7-fold for wheezing), suggesting that a cumulative lifetime exposure to traffic pollutants may raise health risks. Girls showed a greater association between living near a major road and the health outcomes measured, for unknown reasons.

Few studies in the United States have looked at the connection between traffic and the prevalence of childhood asthma, but the results are consistent with emerging evidence from European studies. Smog and other regional pollution is slowly being brought under control by legislation. However, traffic exhaust represents a form of local pollution with public health consequences that is largely unregulated. As a start toward curbing the effects of exhaust, California recently passed a law that prohibits the construction of new schools within 500 feet of freeways. Locating playgrounds, parks, and sports fields a safe distance from busy roads may be another way to prevent children from inhaling exhaust fumes.

## Figures and Tables

**Figure:**
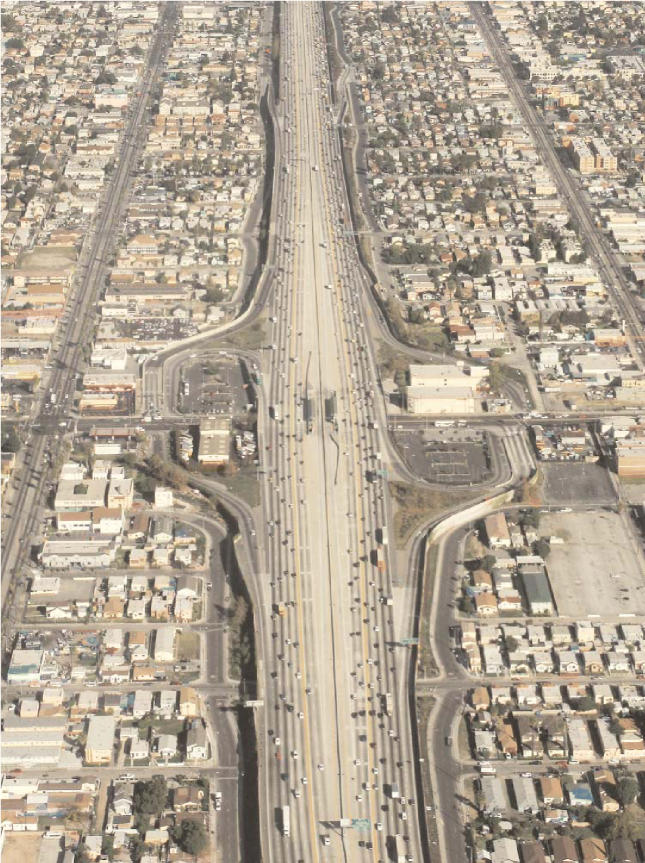
Life on the streets. Children who live within a block of major roads are one and a half times more likely to report asthma or wheezing than those living four or more blocks away.

